# Induction of Autophagy by Pterostilbene Contributes to the Prevention of Renal Fibrosis via Attenuating NLRP3 Inflammasome Activation and Epithelial-Mesenchymal Transition

**DOI:** 10.3389/fcell.2020.00436

**Published:** 2020-06-03

**Authors:** Ying-Jan Wang, Yu-Ying Chen, Ching-Mao Hsiao, Min-Hsiung Pan, Bour-Jr Wang, Yu-Chi Chen, Chi-Tang Ho, Kuo-Ching Huang, Rong-Jane Chen

**Affiliations:** ^1^Department of Environmental and Occupational Health, College of Medicine, National Cheng Kung University, Tainan, Taiwan; ^2^Department of Medical Research, China Medical University Hospital, China Medical University, Taichung, Taiwan; ^3^Institute of Food Science and Technology, National Taiwan University, Taipei, Taiwan; ^4^Department of Occupational and Environmental Medicine, National Cheng Kung University Hospital, Tainan, Taiwan; ^5^Department of Cosmetic Science and Institute of Cosmetic Science, Chia Nan University of Pharmacy and Science, Tainan, Taiwan; ^6^Department of Biotechnology, National Kaohsiung Normal University, Kaohsiung, Taiwan; ^7^Department of Food Science, Rutgers University, New Brunswick, NJ, United States; ^8^Division of Nephrology, Department of Internal Medicine, Chi Mei Hospital, Tainan, Taiwan; ^9^Department of Food Safety/Hygiene and Risk Management, College of Medicine, National Cheng Kung University, Tainan, Taiwan

**Keywords:** autophagy, epithelial-mesenchymal transition, NLRP3 inflammasome, pterostilbene, renal fibrosis

## Abstract

Chronic kidney disease (CKD) is recognized as a global public health problem. NLRP3 inflammasome activation has been characterized to mediate diverse aspect mechanisms of CKD through regulation of proinflammatory cytokines, tubulointerstitial injury, glomerular diseases, renal inflammation, and fibrosis pathways. Autophagy is a characterized negative regulation mechanism in the regulation of the NLRP3 inflammasome, which is now recognized as the key regulator in the pathogenesis of inflammation and fibrosis in CKD. Thus, autophagy is undoubtedly an attractive target for developing new renal protective treatments of kidney disease via its potential effects in regulation of inflammasome. However, there is no clinical useful agent targeting the autophagy pathway for patients with renal diseases. Pterostilbene (PT, trans-3,5-dimethoxy-4-hydroxystilbene) is a natural analog of resveratrol that has various health benefits including autophagy inducing effects. Accordingly, we aim to investigate underlying mechanisms of preventive and therapeutic effects of PT by reducing NLRP3 inflammasome activation and fibrosis through autophagy-inducing effects. The renal protective effects of PT were evaluated by potassium oxonate (PO)-induced hyperuricemia and high adenine diet-induced CKD models. The autophagy induction mechanisms and anti-fibrosis effects of PT by down-regulation of NLRP3 inflammasome are investigated by using immortalized rat kidney proximal tubular epithelial NRK-52E cells. To determine the role of autophagy induction in the alleviating of NLRP3 inflammasome activation and epithelial-mesenchymal transition (EMT), NRK-52E with Atg5 knockdown [NRK-Atg5-(2)] cells were applied in the study. The results indicated that PT significantly reduces serum uric acid levels, liver xanthine oxidase activity, collagen accumulation, macrophage recruitment, and renal fibrosis in CKD models. At the molecular levels, pretreatment with PT downregulating TGF-β-triggered NLRP3 inflammasome activation, and subsequent EMT in NRK-52E cells. After blockage of autophagy by treatment of Atg5 shRNA, PT loss of its ability to prevent NLRP3 inflammasome activation and EMT. Taken together, we suggested the renal protective effects of PT in urate nephropathy and proved that PT induces autophagy leading to restraining TGF-β-mediated NLRP3 inflammasome activation and EMT. This study is also the first one to provide a clinical potential application of PT for a better management of CKD through its autophagy inducing effects.

## Introduction

Chronic kidney disease (CKD) is now known as a major public health issue worldwide (Kumagai et al., 2017). Most forms of CKD are characterized by inflammation and progressive fibrosis in the final stage that eventually affects the structures and functions of kidney leading to an irreversible end-stage renal disease (Kim et al., 2014). Recent attention has focused on the contribution of NLRP3 [nucleotide-binding and oligomerization domain (NOD)-like receptors (NLRs) protein 3] inflammasome activation in the pathogenesis of inflammation and fibrosis in several CKD (Scarpioni and Obici, 2018). In response to damage-associated molecular patterns (DAMPs) such as uric acid (UA), NLRP3 proteins oligomerize, and recruit an ASC (apoptosis-associated speck-like protein containing a C-terminal caspase-recruitment domain), and pro-caspase-1 to complete the formation of the inflammasome (Chen et al., 2016). The inflammasome complexes then induce the caspase-1 activation thereby cleaves the pro-inflammatory cytokines into their active forms such as IL-1β and IL-18 leading to chronic inflammation and cellular damage (Chen et al., 2016). NLRP3 and ASC can be found in renal dendritic cells, infiltrating macrophages, epithelial cells, and tubules (Chang et al., 2014). A recent study indicated that UA could induce NLRP3 expression, caspase-1 activation, IL-1β, and ICAM-1 production in human renal proximal tubule epithelial cells (Xiao et al., 2015). In several CKD disease models, activation of NLRP3 inflammasome, production of IL-1β and activation of P-Smad 2/3 pathways were associated with renal inflammation, epithelial-mesenchymal transition (EMT), and fibrosis (Romero et al., 2017; Qi and Yang, 2018). These studies indicate that downregulation of NLRP3 inflammasome may attenuate renal inflammation and fibrosis; thus, NLRP3 inflammasome may serve as a potential new therapeutic target for CKD.

Recent studies prove the potential role of autophagy in the prevention of CKD (Lin et al., 2019). Autophagy is a highly regulated mechanism that occurs in four phases including induction, nucleation, elongation, and fusion that are tightly regulated by various signaling pathways such as 5′-adenosine monophosphate activated protein kinase (AMPK), autophagy proteins (Atgs) including Atg8 (LC3), Atg5, Atg12, and Atg16L1, and lysosomal enzymes (Chen et al., 2016). Defects in autophagy has been implicated in various diseases and health states, including CKD, neurodegeneration, aging, infectious disease, inflammation, and cancer (Chen et al., 2017b). Currently, autophagy was reported to negatively regulate the NLRP3 inflammasome activation through degradation of NLRP3, ASC, and caspase-1, and pro-inflammatory cytokines as well (Chen et al., 2016). In unilateral ureteral obstruction (UUO)-induced renal fibrosis model, autophagy induction protected fibrosis through regulation of the expression of NLRP3, TGF-β, and IL-1β. Therefore, autophagy is undoubtedly an attractive target for developing new protective treatments of CKD. However, there is no clinical useful agent targeting the autophagy pathway for patients with renal diseases.

Pterostilbene (PT, trans-3,5-dimethoxy-4-hydroxystilbene), a chemical classified as a stilbene, can be found in many foods and herbs such as blueberries, grapes, and tree wood (Chen et al., 2017b). Because of the presence of two methoxy groups, PT exhibits better bioavailability, more lipophilicity, as well as the higher cellular uptake and a longer half-life than its analog resveratrol (Chen et al., 2017b). PT has many health benefits including antioxidant activity, anti-inflammatory effects, anti-obesity, and chemopreventive effects attributed to its unique structure (Lee et al., 2019). Interestingly, PT has been recently demonstrated to effectively protect renal function in a potassium oxonate (PO)-induced hyperuricemia model through regulation of the renal mURAT1, mGLUT9, mABCG2, and mOAT1 (Shi et al., 2012). One other study also suggests that PT has renal protective actions and ameliorates fructose-induced hyperuricemia and fibrosis through suppression of the TGF-β/Smads pathway (Gu et al., 2019). More importantly, PT is now recognized as an effective autophagy inducer in cancer and normal cells (Chen et al., 2017b). The renal protective effects and mechanisms of PT regarding autophagy and NLRP3 inflammasome pathways have not yet been studied. In view of the autophagy inducing effects and the potential beneficial renal protective effects of PT; we try to put a forward a view-point that autophagy induction is a novel mechanism modulating the PT-induced protective effects against CKD. In the present study, we provide the scientific basis of autophagy in the regulation of NLRP3 inflammasome and fibrosis in CKD. Our study also proves that PT could effectively prevent CKD via autophagy contributed to restraining TGF-β-mediated NLRP3 inflammasome activation and EMT in NRK-52E cells. To the best of our knowledge, this is the first study to provide a clinical potential application of PT for a better management of CKD through its autophagy inducing effects.

## Materials and Methods

### Chemicals and Reagents

The uricase inhibitor PO, adenine (AD), and XOD inhibitor allopurinol (AP, urate lowering agent) were purchased from Sigma (St. Louis, MO, United States). TGF-β was obtained from R&D Systems, Inc. (Minneapolis, MN, United States). The NLRP3 inhibitor MCC950, UA assay kit and XOD fluorometric assay kit were purchased from Cayman Chemical Company (Michigan, United States). Pterostilbene (96% purity) was a gift from Sabinsa Corporation (East Winsor, NJ, United States). Primary antibodies against E-Cadherin, fibronectin, α-SMA, and Vimentin were purchased from Genetec Inc. (Montreal, QC, Canada). Antibodies against NLRP3, ASC, caspase-1, IL-1β, AMPK, phosphor-AMPK, mTOR, phosphor-mTOR, p62, LC3-II, and GAPDH were purchased from Cell Signaling (Beverly, MA, United States). The horseradish peroxidase (HRP)-conjugated anti-mouse and anti-rabbit secondary antibodies were obtained from Jackson ImmunoResearch (West Grove, PA, United States).

### Animals

Four-week-old ICR male mice were purchased from BioLASCO Taiwan Co., Ltd (Taipei, Taiwan). They were housed (five mice per cage) in a pathogen-free environment, maintained on Lab Diet 5010 chow (PMI Feeds, Inc, St. Louis, MO, United States) at 24 ± 2°C and 50 ± 10% relative humidity, and subjected to a 12 h light/12 h dark cycle in the Laboratory Animal Center of the National Cheng Kung University Medical College. All animal studies were approved by the Laboratory Animal Center of the National Cheng Kung University Medical College (Approval No. 103293) and performed according to the local guidelines for animal care and protection.

### PO-Induced Hyperuricemia Model

**FIGURE 1:**
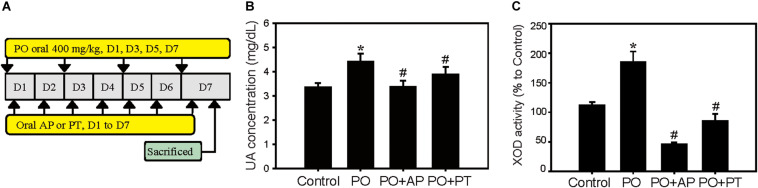
Urate lowering effects of PT in a hyperuricemia animal model. **(A)** Scheme of PO-induced hyperuricemia model as described in the Materials and Methods. **(B)** UA concentration and **(C)** liver XOD activities of mice treated with Control, PO (400 mg/kg), PO + AP (10 mg/kg), or PO + PT (200 mg/kg) for 7 days (*n* = 3 in each group). Data represent the mean ± SD. *p < 0.05 compared with the Control groups. ^#^*p* < 0.05 compared with PO groups. PO: potassium oxonate, PT: pterostilbene, AP: allopurinol, and XOD: xanthine oxidase.

Establishment of the PO-induced hyperuricemia animal model was performed as our previous report (Chen et al., 2017a). Mice were divided into four groups of Control (0.9% normal saline), PO groups (400 mg/kg, hyperuricemia groups), PO + AP (positive treatment groups, AP: 10 mg/kg), or PO + PT (200 mg/kg) groups (Figure 1A).

### XOD Activities of Mouse Livers

Mouse liver XOD activities were analyzed by XOD fluorometric assay kit (Chen et al., 2017a).

### High Adenine Diet-Induced CKD Model

**FIGURE 2:**
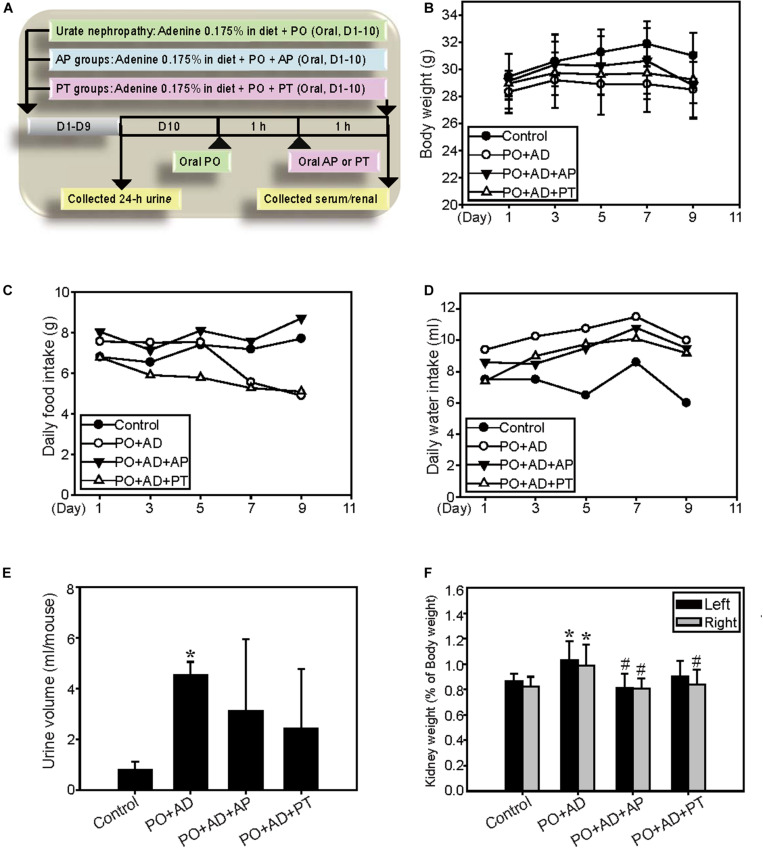
Renal protective effects of PT in a high adenine-induced CKD model. **(A)** The high adenine-induced CKD model was performed as described in Materials and Method. **(B)** Changes of body weight, **(C)** daily food intake, and **(D)** daily water intake are demonstrated for ICR mice fed with 0.9% saline (Control), PO combined with 0.175% adenine (PO + AD), AP (10 mg/kg) combined with PO + AD (PO + AD + AP), or PT (200 mg/kg) combined with PO + AD (PO + AD + PT). Effects of PT on **(E)** 24-h urine output and **(F)** kidney relative weight (kidney weight/final body weight × 100) in mice. Each column and vertical bar are mean ± SD of 5 mice/group. *p < 0.05 compared with the control groups.

The CKD model is adapted by Diwan’s study (Diwan et al., 2013). Briefly, male ICR mice at 5 weeks of age were randomly divided into four experimental groups treated for 10 days (*n* = 5 in each group). The control groups were given powdered mouse food. The PO + AD groups received high adenine diet (0.175% AD in powder) together with orally administration of PO (400 mg/kg/day). The PO + AD + AP and PO + AD + PT groups indicated the mice treated with AP (10 mg/kg) or PT (200 mg/kg) by gastric gavage for 10 days combined with PO + AD (Figure 2A). Urine were collected before the day of sacrificed. All mice were sacrificed 1 h after the last treatment, and blood samples were collected from cardiac puncture for analysis. Kidneys were removed and examined for the morphology at necropsy. The right kidneys were kept in liquid nitrogen for Western blotting analysis, and the left ones were preserved in 10% buffered formalin for immunohistochemistry study.

### UA Analysis

The UA level of serum and urine was determined by the UA kit according to the manufacturer’s instructions as previous reported (Chen et al., 2017a).

### Renal Function Analysis

Mouse serum and urine samples were monitored by the biochemical indices including BUN (blood urea nitrogen), and CRE (creatinine) using commercial kits (FUJIFILM corporation, Tokyo, Japan).

### H&E Staining, Masson Trichrome Staining, and Immunohistochemistry of Kidney Tissues

Serial tissue sections (4 μm) of kidneys were sliced from paraffin-embedded, formalin-fixed kidneys, and stained with hematoxylin and eosin (H&E). For Masson’s trichrome staining, tissues were stained with aniline blue liquid and 1% acetic acid, followed by dehydration with ethanol. Staining showed collagen fibers in blue and cytoplasm in red color. For immunohistochemistry, the slides were incubated with primary and secondary antibodies then developed with the STARRTREK Universal HRP detection Kit (Biocare medical, Concord, CA, United States).

### Cell Culture of Control and Atg5 Knockdown NRK-52E Cells

NRK-52E cells (immortalized rat kidney proximal tubular epithelial cells, CRL-1571) were purchased from Bioresource Collection and Research Center (Food Industry Research and Development Institute, Hsinchu, Taiwan) and were cultured in Dulbecco’s modified Eagle’s medium (DMEM) with 5% fetal bovine serum (HyClone, South Logan, UT, United States), 100 U/ml penicillin, and 100 μg/ml streptomycin (Life Technologies, Inc., Gaithersburg, MD, United States). The cells were treated with TGF-β (2.5 ng/ml), PT (2 μM), or in combination for indicated time points. To establish autophagy defect cells, NRK-52E cells were transfected with the Atg5 knockdown shRNA (TRCN0000327377) which was obtained from the National RNA interference Core Facility located at the Institute of Molecular Biology/Genomic Research Centre, Academia Sinica (Nankang, Taipei, Taiwan). The stable Atg5 knockdown cells [NRK-Atg5(1), (2)] were maintained in DMEM contained with 2 μg/ml puromycin.

### Cell Viability Assay

NRK-52E cells were seeded in a 96-well plate at a density of 1 × 10^4^ cells/well for overnight then treated with TGF-β (2.5, 5, and 10 ng/ml), PT (0.5, 1, 2 μM), or TGF-β combined with PT for 24, 48, or 72 h and the viability was detected by MTT assay (Chen et al., 2010).

### Detection of Autophagy by Flow Cytometry

NRK-52E and Atg5 knockdown cells were treated with TGF-β (2.5 ng/ml), PT (2 μM), or in combination for indicated time points and autophagy was detected by acridine orange (AO) staining and analyzed by flow cytometric analysis (FACScan, Becton Dickinson, San Jose, CA, United States) and quantified using FlowJo 7.6.1. software (Tree Star Inc, Ashland, OR, United States; Chen et al., 2010).

### Western Blot Analyses

Randomized frozen kidney samples or NRK-52E cells from different treatment groups were homogenized and lysates were subjected to gel electrophoresis and immunoblotting (Chen et al., 2017c). Immunoreactive proteins were visualized with a chemiluminescent detection system (PerkinElmer Life Science, Inc. MA, United States) and BioMax LightFilm (Eastman Kodak Co., New Haven, CT, United States) according to the manufacturer’s instructions.

### Immunofluorescence Staining

After treatment with TGF-β, PT, or in combination for 72 h, cells were fixed, washed then incubated with primary and fluorophore-conjugated secondary antibodies for 1 h, mounted with Vectashield Mounting Medium with DAPI (Vector Laboratories, #H-1600, Peterborough, United Kingdom), and then analyzed using a fluorescence microscope.

### Statistical Analysis

Results are expressed as mean ± standard error of the mean (SEM). Experimental data were analyzed using the Student’s *t* test. Differences were considered to be statistically significant when the *p* value was less than 0.05.

## Results

### Urate-Lowering Effect and XOD Inhibition Activity of PT in the Hyperuricemia Animal Model

Previous studies indicated that UA is the main risk factor for CKD (Kumagai et al., 2017). Accordingly, we first verify the urate-lowering effect of PT in the established PO-induced hyperuricemia model (Figure 1A). The results showed that PO significantly increased the UA concentration level (4.63 ± 0.32 mg/dL) when compared to the control groups (3.35 ± 0.19 mg/dL; Figure 1B), whereas PT administration followed by PO (PO + PT groups) effectively decreased UA levels (3.90 ± 0.31 mg/dL) compared to the PO groups. AP (therapy control) also significantly lowered serum UA levels in mice (PO + AP groups, 3.00 ± 0.23 mg/dL; Figure 1B). XOD activity was assayed using mouse livers and the inhibition of XOD activity by AP and PT showed 60.1% and 25%, respectively (Figure 1C). The results suggest the urate lowering effects of PT could partially depend on XOD inhibitory effects.

### PT Reduced Renal Damage and Fibrosis in the High Adenine-Induced Renal Fibrosis Model

**FIGURE 3:**
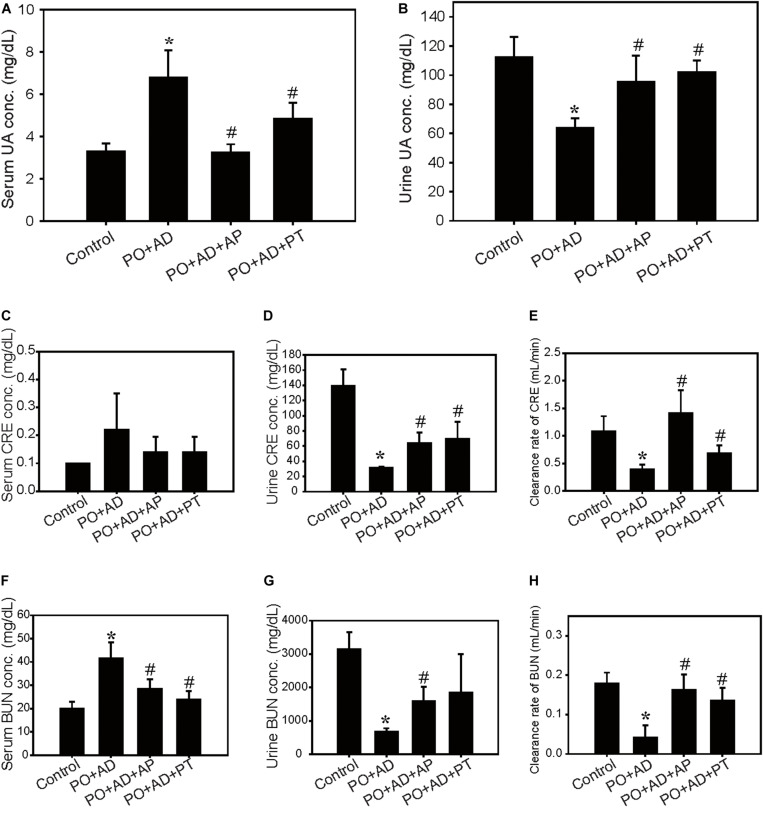
Improvement of renal function by PT in the CKD model. Serum and urine levels of UA **(A,B)**, creatinine (CRE; **C,D)**, blood urea nitrogen (BUN; **F,G)**, and clearance rate of CRE **(E)**, and BUN **(H)** in the CKD model. Data were expressed as mean ± SD of 5 mice per group. *p < 0.05 compared with the control groups. #*p* < 0.05 compared with PO + AD groups.

The renal protective mechanisms of PT were further investigated in the high AD diet-induced CKD model which has been reported related to the pathogenesis in human CKD (Figure 2A). Concomitant administration of PO and AD (PO + AD) reduced daily food intake and the final body weights in mice (Figures 2B,C) but increased water intake and urine output (Figures 2D,E). On the contrary, PT mitigates these actions as shown in Figures 2B–E. The weight of kidneys showed significantly increased in PO + AD groups, whereas PT prevents increased in kidney weights as shown in Figure 2F. Moreover, renal function analysis indicated the protective effects of PT comparted with PO + AD groups that mice treated with PT 200 mg/kg for 10 days (PO + AD + PT groups) decreased serum UA, CRE, and BUN levels compared to PO + AD groups (Figures 3A,C,F). Simultaneously, PT increased urine UA, CRE, and BUN levels (Figures 3B,D,G), and the clearance rate of CRE, and BUN as well (Figures 3E,F). Similar results were found in AP treated groups further confirmed the renal protective effects of PT.

**FIGURE 4:**
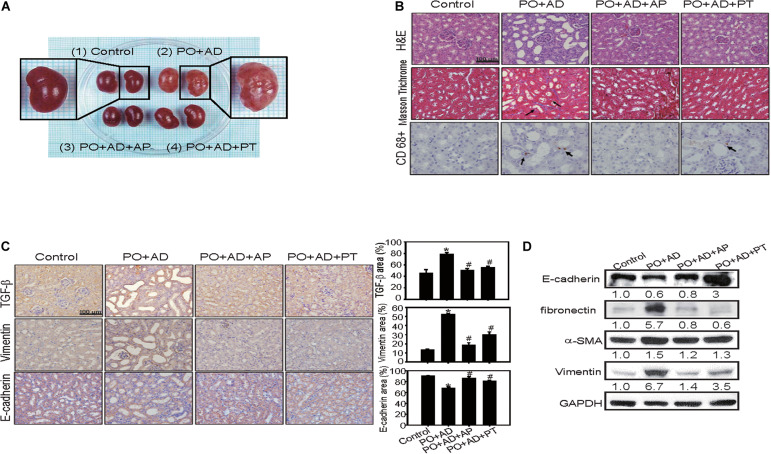
PT alleviates inflammation and interstitial fibrosis in kidney tissues in the CKD model. **(A)** The size of kidney was increased in PO + AD groups. The appearance of kidney in PO + AD groups was rough and pale. **(B)** Renal tubular injury was assessed by H&E staining. The deposition of fibrosis in renal tissues was determined by Masson Trichrome staining. Blue color represents collagen fibers, red color represents muscle fibers. Infiltration of macrophages was detected by CD68 staining (brown color, arrow indicated). **(C)** Tubulointerstitial inflammation and EMT was assessed by staining of TGF-β, Vimentin, and E-cadherin. The results of immunohistochemistry were quantified by ImagJ (*n* = 3). Bar = 100 μm. *p < 0.05 compared with the control groups. #*p* < 0.05 compared with PO + AD groups. **(D)** Compared with PO + AD groups, PT increased the expression of E-cadherin, and decreased the expression of fibronectin, α-SMA, and Vimentin in renal tissues detected by Western blotting analysis. GAPDH was used as an internal control. Representative data from one of three independent experiments are shown. The number below each line indicates the relative intensity of protein expression compared to the control (defined as 1; Figures 4–7).

Renal morphology examination showed critical roughness morphology in PO + AD groups compared with PT or AP treated groups (Figure 4A). H&E and immunohistochemistry staining further revealed obvious tubular-interstitial damage with tubular dilation, interstitial fibrosis (Masson Trichrome staining), infiltration of inflammatory cells (macrophages, CD68^+^), and focal tubular atrophy in PO + AD groups (Figure 4B). With the treatment of PT, tubular interstitial damage, fibrosis, and infiltration of inflammatory cells were significantly alleviated (Figure 4B). Given the prominent role of TGF-β in renal fibrosis by inducing EMT leading to promotion of tubular epithelial cells to myoblast (Wang et al., 2015), the results of immunohistochemistry demonstrated that PT administration significantly reduced TGF-β expression compared to PO + AD groups (Figure 4C). EMT is characterized in which tubular cells loss the epithelial phenotypes such as E-cadherin, and acquire new characteristic features of mesenchymal properties such as ZO-1, Vimentin, fibronectin, and α-SMA (Bai et al., 2014). The results of immunohistochemistry and Western blotting analysis showed that PT suppressed the expression of fibronectin while increased the expression of E-cadherin compared with PO + AD groups (Figures 4C,D). Taken together, the results of *in vivo* study strongly implicated the renal protective effects of PT, through the inhibition of UA synthesis, reducing inflammation, downregulation of TGF-β mediated EMT pathways and fibrosis (Figures 1–4).

### PT Inhibits TGF-β-Induced EMT and Inflammasome Activation in NRK-52E Cells

**FIGURE 5:**
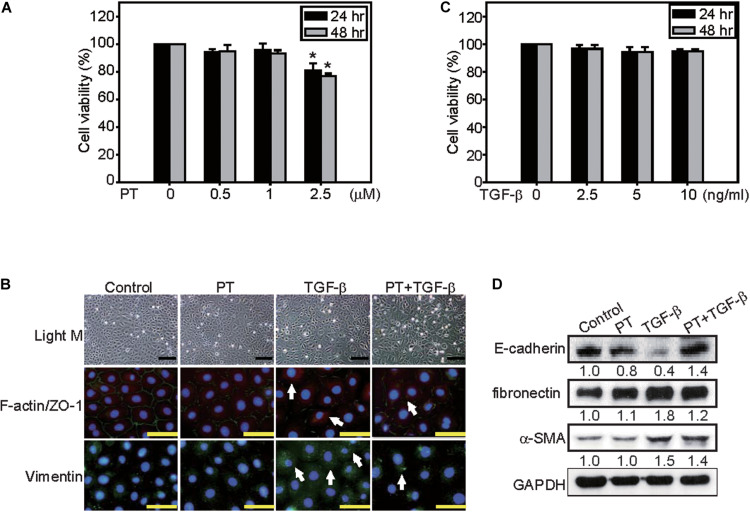
PT inhibits TGF-β-triggered EMT in NRK-52E cells. NRK-52E cells were treated with **(A)** 0, 0.5, 1, or 2.5 μM PT, or **(B)** 0, 2.5, 5, or 10 ng/ml TGF-β for 24 or 48 h. Cell viability were detected by MTT assay. Mean ± SD; *n* = 3. **(C)** NRK-52E cells were treated with DMSO (Control), PT 2 μM, TGF-β 2.5 ng/ml, or PT combined with TGF-β for 72 h. The morphological changes of NRK-52E cells were recorded under a phase-contrast microscopy. F-actin/ZO-1 co-staining, or Vimentin staining were determined by immunofluorescence staining. Bar = 100 μm **(D)** The expression of E-cadherin, fibronectin, α-SMA, and Vimentin were determined by Western blotting analysis in NRK-52E cells treated with PT, TGF-β, or PT + TGF-β. The membrane was probed with anti-GAPDH to confirm equal loading of proteins. Immunoblots are representative of at least three independent experiments.

The protective mechanisms of PT in alleviating fibrosis was further investigated in NRK-52E cells. Treatment with PT or TGF-β at lower concentration for 24 or 48 h did not induce cytotoxicity in NRK-52E cells (Figures 5A,B). However, treatment with TGF-β for 72 h, many NRK-52E cells became spindle fibroblast-like cells, meanwhile, the expression of fibronectin, ZO-1, and α-SMA were increased (Figures 5C,D). In contrast, pretreatment of PT for 1 h significantly attenuated TGF-β-induced EMT characteristics in cells (Figures 5C,D).

**FIGURE 6:**
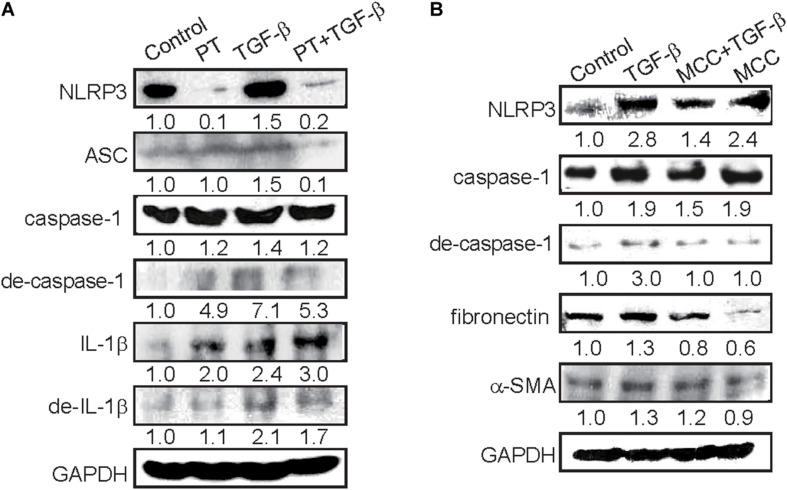
PT and MCC950 attenuates TGF-β-induced NLRP3 inflammasome activation and EMT in NRK-52E cells. **(A)** Immunoblotting for NLRP3 inflammasome components NLRP3, ASC, caspase-1, degraded form of caspase-I, IL-1β, and degraded form of IL-1β in NRK-52E cells following treatment with DMSO (Control), PT 2 μM, TGF-β 2.5 ng/ml, or PT combined with TGF-β for 48 h (PT + TGF-β). **(B)** Immunoblotting for NLRP3, ASC, caspase-1, degraded form of caspase-1, and EMT markers fibronectin and α-SMA at 48 h following treatment with DMSO (Control), TGF-β 2.5 ng/ml, TGF-β combined with NLRP3 inhibitor MCC950 (MCC 10 nM), or MCC in NRK-52E cells. The membrane was probed with anti-GAPDH to confirm equal loading of proteins. Immunoblots are representative of at least three independent experiments.

A previous study suggested that NLRP3 inflammasome activation in response to TGF-β could contribute to EMT, however, the role of NLRP3 inflammasome in EMT regulation is still obscured (Zhang et al., 2020). Then we determine the effects of PT on the regulation of NLRP3 inflammasome and found that NLRP3 expression increased in response to TGF-β, but significantly inhibited by the presence of PT (Figure 6A). Consequently, the expression of ASC and degraded form of caspase-1 were decreased leading to the reduction of maturation form of IL-1β after PT treatment (Figure 6A). Further, pretreatment with a potent NLRP3 inhibitor MCC950 significantly reduced the expression of NLRP3, caspase-1 activation, followed by reduced EMT markers including fibronectin and α-SMA expression, in which confirmed the role of NLRP3 inflammasome in EMT driven by TGF-β (Figure 6B). The results strongly implicated that PT downregulates NLRP3 inflammasome activation and consequently inhibits TGF-β-mediated EMT in NRK-52E cells.

### PT Inhibits NLRP3 Inflammasome Activation by Augmenting Autophagy

**FIGURE 7:**
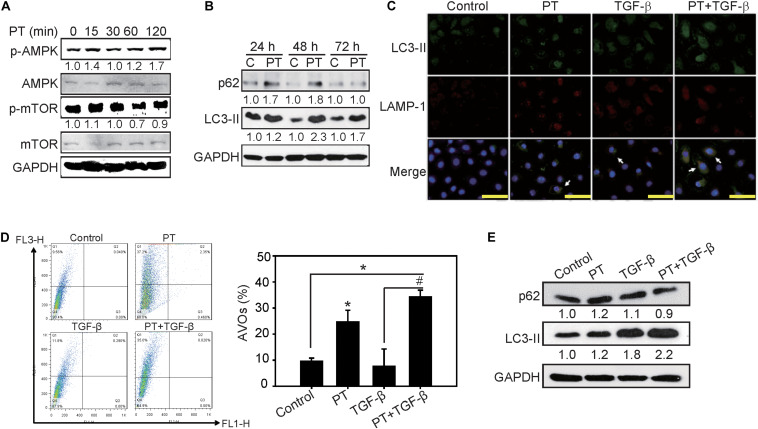
Autophagy inducing effects of PT in NRK-52E cells. **(A)** PT 2 μM induced activation of AKT followed by inhibition of mTOR pathways in a time-dependent manner. **(B)** Autophagic markers p62 and LC3-II were determined using Western blotting analysis in NRK-52E cells treated with PT for 24, 48, and 72 h. **(C)** Immunofluorescence staining showed the autolysosomes co-stained with LC3-II and lysosomal marker LAMP-1 in PT treated groups (arrow indicated). Bars = 100 mm. **(D)** The induction of autophagy was measured by acidic vesicular organelles (AVOs) using flow cytometry. Quantification of AVOs in NRK-52E cells treated with DMSO (Control), PT 2 μM, TGF-β 2.5 ng/ml, or PT combined with TGF-β for 48 h. The data represent the means ± SD of three independent experiments; *P < 0.05 compared with Control groups; and ^#^*P* < 0.05 compared with TGF-β groups. **(E)** Immunoblotting for autophagy markers p62, and LC3-II in NRK-52E cells followed by the treatment of DMSO (Control), PT 2 μM, TGF-β 2.5 ng/ml, or PT + TGF-β for 48 h. The membrane was probed with anti-GAPDH to confirm equal loading of proteins. Immunoblots are representative of at least three independent experiments.

**FIGURE 8:**
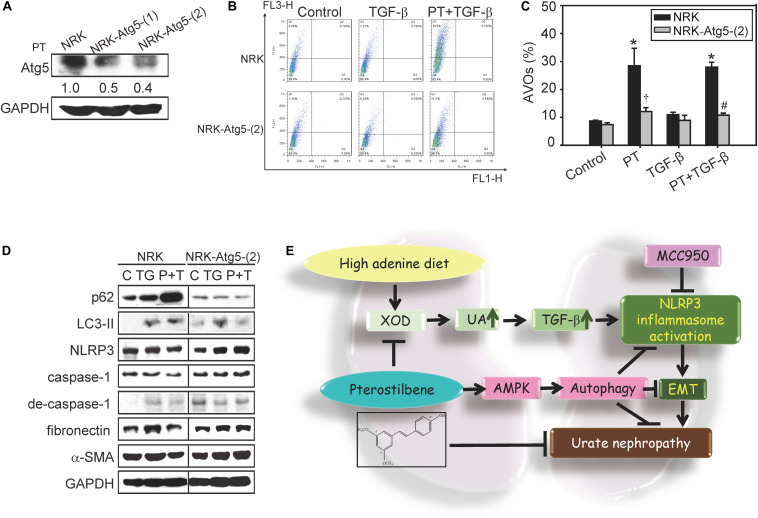
Role of autophagy in attenuating NLRP3 activation and EMT in response to PT treatment. **(A)** Atg5 expression in NRK-52E cells stably transfected with Atg5 knockdown shRNA [Atg5-(1) and Atg5-(2)]. **(B)** The induction of autophagy by PT was measured by flow cytometry in NRK-52E and Atg5 knockdown NRK-Atg5-(2) cells. The data represent the means ± SD of three independent experiments; *P < 0.05 compared with Control groups; ^#^*P* < 0.05 compared with NRK-52E cells. **(C)** The percentage of acridine orange positive staining cells in NRK and NRK-Atg5-(2) treated with PT (2.5 μM), TGF-β, or PT + TGF-β for 48 h. **(D)** The protein expression of autophagy markers (p62 and LC3-II), NLRP3 inflammasome components (NLRP3, caspase-1 and degraded form of caspase-1), and EMT markers (fibronectin and α-SMA) were detected by Western blotting in NRK and NRK-Atg5-(2) cells following 48 h treatment with TGF-β (TG) or PT combined with TGF-β (P + T). The membrane was probed with anti-GAPDH to confirm equal loading of proteins. Immunoblots are representative of at least three independent experiments. **(E)** Proposed model for renal protective effects of PT. PT significantly reduces UA production and XOD activities, prevents renal dysfunction, and ameliorates renal fibrosis in animal models. The mechanistic studies implicate that PT induces autophagy through AMPK activation to restrain TGF–triggered NLRP3 inflammasome activation and EMT, subsequently contributing to the protection of renal fibrosis.

Previous reports indicated that autophagy acts as a negative regulator to restrain NLRP3 activation from reducing the expression of inflammasome components (Chen et al., 2017b). To clarify whether PT downregulates NLRP3 inflammasome activation through autophagy induction, AMPK, and mTOR signaling pathways were examined firstly. PT time-dependently activates AMPK, leading to mTOR inhibition and autophagy induction as evidenced by LC3-II induction and p62 expression in NRK-52E cells (Figures 7A,B). To further confirm the complete autophagic flux induced by PT, the results of immunofluorescence staining showing that co-localization of LC3-II and the lysosome marker LAMP-1 could be observed in PT alone and PT + TGF-β treated groups (Figure 7C). In addition, the results of AO staining and Western blotting analysis further confirmed autophagy inducing effect of PT in NRK-52E cells (Figures 7D,E). To gain insight the role of autophagy in downregulation of NLRP3 inflammasome and the subsequent EMT pathways, NRK-52E cells with Atg5 knockdown [NRK-Atg5-(1) and (2)] was used. The expression of Atg5 (Figure 8A) was partially reduced in accordance with reduced autophagy after treated with PT (Figures 8B,C). Compared to NRK-52E cells, Atg5 knockdown cells [NRK-Atg5-(2) cells] revealed a significant increased expression of NLRP3, caspase 1, fibronectin, and α-SMA followed by the treatment with PT combined with TGF-β for 72 h (Figure 8D). Collectively, these results implicated the significant role of autophagy induced by PT in preventing NLRP3 inflammation activation and subsequently EMT in NRK-52E cells.

## Discussion

Chronic kidney disease is recognized as a global public health problem. Evidence has demonstrated that UA plays a crucial role in the CKD and using UA-lowering drugs may represent an effective therapeutic venue for delaying CKD progression (Kumagai et al., 2017). However, at present, the urate lowering agents such as AP or probenecid still possess a number of adverse side effects including allergic and hypersensitivity problems, nephropathy, and liver toxicity (Chen et al., 2017a). Therefore, the development of the effective and safety strategy at lowing UA thereby alleviating CKD is still necessary. In this study, we demonstrate a novel underlying urate lowering mechanisms and preventive effects of PT in renal inflammation and fibrosis. As shown in Figure 8E, high adenine treatment promotes urate nephropathy characterized by renal inflammation and fibrosis that is mainly regulated by TGF-β production and NLRP3 inflammasome activation. PT is effective in inhibiting XOD activity, reducing UA production, inflammation, and consequently renal fibrosis in mice. At molecular levels, PT induces autophagy leading to restraining TGF-β-mediated NLRP3 inflammasome activation and EMT in NRK-52E cells. To the best of our knowledge, this is the first report investigating the protective effects of PT in CKD by attenuating NLRP3 inflammasome activation and EMT via autophagy induction.

Our previous study was the first to demonstrate that PT is a unique autophagy inducer (Chen et al., 2010). Through its autophagy inducing effects, PT could act as a promising protective agent for autophagy-defective diseases (Chen et al., 2017b). Notably, autophagy has a great impact on the maintenance of renal functions and homeostasis (Lin et al., 2019). For instance, autophagy has renal protective effects on the proximal tubular cells during acute kidney injury (AKI) in response to hypoxia and ROS (Wang et al., 2012). In addition, autophagy takes places in podocytes, mesangial cells, and tubular cells that helps repair and regenerates damaged kidneys (Lin et al., 2019). Impaired autophagy in kidneys resulted in podocyte loss, massive proteinuria, inflammation, and renal interstitial fibrosis in several CKD models (Lin et al., 2019). Xu et al. (2016) also indicated that autophagy defects can directly lead to excessive deposition of extracellular matrix such as fibronectin or indirectly activate renal fibrosis by enhancing oxidative stresses. Importantly, clinical studies indicated that patients with CKD have altered autophagy response (Chen et al., 2013). Our results also showed that depletion of autophagy by knockdown of Atg5 gene leads to increase the expression of NLRP3, degraded form of caspase-1, fibronectin, and α-SMA (Figure 8D). These studies indicated that autophagy induction is now considered an effective therapy for AKI or CKD but there is no clinical useful agent targeting the autophagy pathway for patients with renal diseases (Lin et al., 2019). As a result, our results firstly indicated that PT could act as an autophagy inducer resulting in downregulation of inflammasome and subsequent EMT thereby alleviating CKD (Figure 8).

In addition to autophagy regulation in CKD, NLRP3 inflammasome activation has been characterized to mediate diverse aspect mechanisms of urate nephropathy through regulation of proinflammatory cytokines, tubulointerstitial injury, glomerular diseases, renal inflammation, and fibrosis pathways (Mulay et al., 2018). In the process of fibrosis, the tubular epithelial cells have the capacity to acquire an EMT in the injured kidney through the most important factor TGF-β (Chuang et al., 2013). A previous study of Wang et al. (2013) indicated that NLRP3 is required for optimal TGF-β signaling and its downstream P-Smad activation. Zhang et al. (2020) also indicated that TGF-β-mediated NLRP3 inflammasome activation may induce HMGB1 release and Gasdermin D cleavage contributing to renal fibrosis in Ang II-induced CKD. However, the regulation of NLRP3 in TGF-β mediated EMT process is largely unclear so far. In the present study, we demonstrated that TGF-β acts as a priming signal leads to NLRP3 protein induction, and the activated signal leads to caspase-1 activation and the following EMT (Figure 5). The role of NLRP3 in EMT is then confirmed by pretreatment with NLRP3 inhibitor MCC950. The results further implicated the protective role of PT in mitigating TGF-β induced EMT could be mediated by downregulation of NLRP3 protein expression and subsequently inhibition of IL-1β production (Figure 6A). Consistent with our findings, Nam et al. indicated that autophagy attenuates tubulointerstitial fibrosis through regulation of the expression of TGF-β, IL-1β, and NLRP3 inflammasome activation in UUO model. Therefore, we suggest that PT is effective in the prevention of NLRP3-inflammasome-mediated inflammation and EMT via autophagy.

As mentioned above, PT has attracted more attention for its beneficial health effects compared to its analog resveratrol because of its unique structure (Sun et al., 2016). PT has diverse pharmacological benefits for the prevention and treatment of variety of diseases such as cancer, diabetes, cardiovascular disease, dyslipidemia, and inflammation (Chen et al., 2018). In the present study, we are also the first one to demonstrate that PT could inhibit XOD activities that exerts as one of the urate lowering mechanisms of PT. XOD is a rate limiting enzyme for UA production and ROS production. Similar to our findings, a previous study also highlighted that flavonols such as quercetin had an inhibitory effect on XOD activities therefore modulate the activities of superoxide dismutase (Majo et al., 2014). These studies suggest the beneficial health effects of natural products in modulating of enzymes responsible for ROS production and inflammation (Majo et al., 2014). In clinical trials, PT could inhibit of PGE2 activity and reduce blood pressure in subjects (Riche et al., 2013). The results of clinical trials where the subjects took PT daily for 8 weeks with no serious adverse events indicated that PT is generally safe for use in humans up to 250 mg/day (Riche et al., 2013). In a preclinical study, mice fed with PT for 28 days at a dose up to 3000 mg/kg, equivalent to 50 times of human intake (250 mg/day) showed no significant toxic effects or adverse biochemical parameters compared to control (Lee et al., 2019). Consequently, PT is an attractive candidate for management of various diseases including CKD because of its low toxicity and wide health benefits. Taken together, we have provided a new evidence for a better understanding of the relationship between autophagy induction and NLRP3 inflammasome activation by PT in CKD models. These findings implicate that PT could be a novel preventive and therapeutic agent for CKD because of its ability of inhibiting NLRP3 activation and EMT by autophagy induction.

## Data Availability Statement

The raw data supporting the conclusions of this article will be made available by the authors, without undue reservation, to any qualified researcher.

## Ethics Statement

The animal study was reviewed and approved by the Laboratory Animal Center of the National Cheng Kung University Medical College (Approval No. 103293).

## Author Contributions

R-JC, Y-YC, and C-MH carried out the animal studies, cell culture, drug treatment, flow cytometry analysis, immunohistochemistry, western blot analysis, and performed the statistical analysis. C-TH provided pterostilbene. Y-CC and K-CH precipitated in the animal and project submission. R-JC and Y-JW participated in the sequence alignment and drafted the manuscript. R-JC, B-JW, and M-HP participated in the design of the study and coordination and helped to draft the manuscript. All authors read and approved the final manuscript.

## Conflict of Interest

The authors declare that the research was conducted in the absence of any commercial or financial relationships that could be construed as a potential conflict of interest.
